# Emotional Well-Being of Parents Undergoing Family Therapy in a Children’s Psychiatric Clinic

**DOI:** 10.1192/j.eurpsy.2022.1081

**Published:** 2022-09-01

**Authors:** D. Dovbysh, M. Bebchuk, I. Kopytina, A. Rodionova, I. Maruk, I. Balyakina

**Affiliations:** 1 Scientific-Practical Сhildren’s and Adolescents Mental Health Center n.a. G. Sukhareva, Moscow Department of Health Care, Clinical Psychology, Moscow, Russian Federation; 2 Federal State Autonomous Educational Institution of Higher Education I.M. Sechenov First Moscow State Medical University under the Ministry of Health of the Russian Federation (Sechenov University), Pedagogy And Clinical Psychology, Moscow, Russian Federation

**Keywords:** family therapy

## Abstract

**Introduction:**

Parents today can be important members of a multi-professional team, helping children with mental illness. The well-being of the parents is an important factor in successfully helping the child and willingness to cooperate with specialists.

**Objectives:**

To investigate the experiences of parents undergoing family psychotherapy on an outpatient basis and during a child’s hospitalization.

**Methods:**

86 parents who applied for family therapy on an outpatient basis and 80 parents (main group) of hospitalized children took part in the study. Participants were offered the following questionnaires: Beck Hopelessness Scale, modified scales of the Dembo-Rubinstein, GAD-7, PHQ-9, Quality of Life Enjoyment and Satisfaction Questionnaire. The study was conducted from 04/01/2021 to 04/14/2021.

**Results:**

The main group significantly differs from the outpatient group in the following parameters (according to the t-test): the level of depression (M=18,34 and M=11,61 respectively) and anxiety (M=12,07 and M=7,96 respectively), the quality of life in the sphere of emotional well-being, social sphere, activity and free time, as well as the happiness self-assessment scales. The results on the scales of depression and hopelessness are inversely significantly associated with the willingness of parents to participate in family psychotherapy (r=-0,74, p=0,01) visit the child (r=-0,58, p=0,05), and regularly contact a doctor (r=-0,61, p=0,05).

**Conclusions:**

Depending on the well-being of family members and the tasks facing the family, family assistance may differ depending on the stage of treatment of the child.

**Disclosure:**

No significant relationships.

